# Address before the Alumni Association of Rush Medical College, Feb. 2, 1870

**Published:** 1870-03

**Authors:** A. E. Ames

**Affiliations:** Minneapolis, Minn., President


ARTICLE III.—*Address before the Alumni Association of Rush Medical College*, February 2, 1870. By A. E. AMES, M.D., of Minneapolis, Minn., President.* Published by request of the Association.

GENTLEMEN: Called by the voice of the Alumni of the Rush Medical College to this chair, emotions of sincere thanks well up from my soul for the distinguished favor conferred.

By the rules of our association, the president is required to give an address.

> "Speech is morning to the mind;
> It spreads the beaut ous images abroad
> Which else lie furled and clouded in the soul."

It is customary on occasions like this, to congratulate and to welcome your return home. Friends and brothers greet you with the warm grasp of the hand; and, with joyous hearts, enlivened with thoughts of the pleasing memories of the past, you have met here to renew acquaintance, to revive in memory the pleasant days in college—to tell each other your trials, and of your professional life made since you left dear old Rush.

How oft have our minds returned to college days, to call up in memory the pleasant intercourse with students, our relations with teacher, the kindness of treatment, the courteous conduct and pleasant demeanor—each have joyed in such remembrances.

Those days with each of us were so full of thought and aspiration. Before each stood the future as unknown; the periphery, like a faint line of light, quickened hope for a profitable position—for good friends, for wealth, and for happiness.

How earnestly did our beloved professors labor to instruct us in the various branches of a medical education. Perhaps none

* Dr. Ames was the first graduate of the College.

of us have forgotten their warnings and advice given when we went forth from this college to make a life in our profession.

The notes of my journey before me lie:

> "I turn the leaves back faintly,
> The pictures of the past arise;
> Strange forms go by—in memory
> Sweet voices whisper."

In early life, for a short time your speaker was permitted to sit at the feet of a gifted teacher. Since that time, for a period of twenty-six years, he has lived on the frontier—away toward the setting sun, where there was but little to refine in the moral way; and yet in that district Nature was young and very beautiful. That locality has changed, and it is now fully under the influences of industry—of the arts and sciences; onward to scenes more glorious.

On this occasion it may be profitable to turn our minds back for a few years. The achievements of the past have been wonderful—change has rapidly succeeded change for possession and riches. The fair fields of Nature—the prairies of the Northwest that once were so gorgeously ornamented with violets and flowers—now yield golden harvests of grain in great profit to the husbandman. The narrow, winding foot-path made by the red man has been lost, since the railroad has been built, which is now our highway of travel and for commerce along the valleys, across the plains, and over the mountains—and the slow penny-post and coach, through storm, sunshine and mud, at the speed of twenty miles a day; now the mails are transmitted by railway, hundreds of miles a day, and communications are sent by lightning—*instanter*. Many of us have witnessed the adaptation of physical forces, during these decades, to supply the wants of man. We have lived in a famous day of improvements. Our hearts are glad, and our souls are astonished in beholding the great changes.

> "A mighty realm is waking at our feet
> —— to life and beauty,
> —— where millions shall meet,
> And Peace shall reign in majesty sublime."

Chicago, in the year 1836, was a town of 3,000 inhabitants, streets without bottoms, with a cosmopolitan or a heterogeneous composition of races of mankind. Her position was right, the country south, west and north of this locality of the best in virginity on the face of the globe. As the fair land began to change from an uncultivated to a cultivated state, so the city of Chicago began to develop, commerce began to whiten the waters of the great chain of lakes with laden wealth from her port, the railroads soon reached forth to connect her with other cities and districts, and day by day, and year by year, Chicago has astonished the wide world with her growth and commercial strength. Chicago is now a city of 300,000 inhabitants — a marvel to the world. Those who have had doubts in regard to her development have rolled up their skeptical maps and mathematical errors and laid them one side. Chicago is the centre of trade for a great agricultural and manufacturing district — a commercial centre — a home for the fine arts — the seat of education. She bears these honors with becoming grace. Behold her palatial residences, fragrant gardens, ornamental parks, public grounds, streets and boulevard walks — all so beautiful. The public bridges, water and gas works, driveways, balconies, and pavilions, are ornamental and useful; the productions of an intelligent, a wise, and a live people.

> "The school-house and church rose side by side."
> "Thus moves the tide of great events."

In this unparalleled growth of a city and the development of a country, it is of interest to know and realize that the moral and intellectual faculties of the people have kept pace; that the arts and sciences have lent their strength in the great change; and the virtue and peace of the inhabitants have been strengthened; home, the place of rest, made more secure.

The question is often asked, have these glorious achievements in the arts and sciences — the building strong cities and the developing a nation of nations during the last quarter of a century — resulted in great good and happiness for our race? or have our people been weakened in their moral and physical forces? The evidences are of their great ability. Perhaps you may inquire, how is it with the profession of medicine? and what are the evidences of the onward progress of the science of medicine? Time will fail to mention many of the evidences of the achievements of the science of medicine made during the last twenty-six years. There are a few persons present here to-day who listened to the first medical lectures given by the lamented Brainard in this city, in his office on South Clark street, in the winter of 1841 and 1842, on Anatomy, Physiology, and Surgery, while he was Professor in the St. Louis University.

The first regular course of lectures of the Rush Medical College was commenced in two rooms on South Clark street, to a class of twenty-two students, December, 1843. Prof. Daniel Brainard, the President of this College, held two chairs; Prof. James V. Z. Blaney (now President) held two chairs; Prof. John McLean one, and Prof. M. L. Knapp one chair. This was the beginning — the lectures were good, never better given in any amphitheater. Since that time great changes have taken place in Professors, buildings and conveniences. Wise and good men have been called as teachers, to wit: Profs. John Evans, Austin Flint, Graham N. Fitch, Wm. B. Herrick, Joseph W. Freer, J. Adams Allen, Ephraim Ingals, DeLaskie Miller, R. L. Rea, Edwin Powell, Moses Gunn, Joseph P. Ross, and Edwin L. Homes, all qualified to teach; gentlemanly, social and kind — *a tower of strength.* To-day it is a proud position to fill either chair in this College.

No College promises more to the student than the Rush Medical College. In an early day its influence was felt throughout the sparsely settled Northwest. Many persons at that time were practicing medicine of a necessity, without a diploma from any institution, or sufficient qualifications. Many of those were men of good character and great energy. They were in the range of the influence of this College. These men were by the regulations of this College induced to come in and attend lectures, four years of reputable practice having been allowed as the equivalent of one course of lectures. Some attended but one course and graduated respectably; but the majority, of their own accord, convinced of their deficiences, attended a second, and some even a third course, before presenting themselves as candidates for the honors of this Institution.

This regulation, though objected to by some at the time, proved to be in the end an efficient means for the elevation of the standard of attainments among the profession of our country. Each of you will remember the strict and rigid examination you were required to pass in order to obtain your diploma; in your practice, the profit of attending lectures here — especially if you have practiced medicine alongside of other medical gentlemen who were educated elsewhere. Here you became acquainted with the phases of diseases peculiar to this geographical division of our common country, and how to treat them.

It is a fact, go where you please in the medical colleges on the Atlantic and the Pacific coasts, and of the cities of the Middle States, you can not find a more appreciative class than the one in attendance here this session, nor can you find better clinical teaching in any college in America than in this.

Again we are proud to say, that those of our Alumni who were surgeons in the great rebellion, acquitted themselves with honor. None performed better service, as can be proven by their clear records in the office of the Surgeon-General of the United States army.

Since the organization of this college about 1,400 students have received honors of graduation.

The first college building cost $3,500, and was occupied Nov. 5, 1845, and was used during the course of lectures for the session of 1845 and 1846. At that session the Faculty was full.

Soon that building was found to be too small. In the summer of 1854, the first building was enlarged at the expense of $10,000.

During the summer of 1867, the old building was found insufficient; and this elegant edifice was erected and dedicated with appropriate ceremonies to *Science and Humanity*, October 2, 1867.

Gentlemen: This, as a college building, is unequaled in America — an evidence that the science of medicine is keeping pace with other signal developments.

Perhaps you, my kind friends, may not think it wrong for me to say a few words of him whose fame has gone into all lands; that Professor Daniel Brainard's foresight and great energy did more than any one man to build up this College. My tongue was never learned to speak eloquent words, or to pronounce obituaries for the great and good, yet for the love we bear to his name, it is my privilege to award great credit to his memory — to the brilliant operator — the surgeon. He was the founder of the Rush Medical College. This edifice is a monument to his labors, of his attainments, his faithfulness to the principles of his profession and of his renown.

None who ever heard him speak can forget his fervency in address—his gentlemanly and courtly manners—ever kind, genial, and true—an example to all who would live the life of a manly man and a teacher.

This stately edifice is his monument—ye are his building. " He died like a man, and fell like one of the princes." The poet Dowler well said of *him* whose name is so dear to science :

" In labors great, in knowledge deep,
His work well done—let Brainard sleep;
Erect his tomb hard by the lake,
Where waves on waves resounding break,
And chant upon the shelly shore
His requiem, for ever more."

Another of those whose labors in the earlier days of this College contributed largely to its success, has passed away from earth. A generous and social gentleman, an enthusiastic student and teacher, beloved by all who knew him, was the late William B. Herrick. As an anatomist he had but few equals. As a teacher of physiology, his expansive mind could not be confined within the narrow limits of the books. His acute perceptions, and his unusual powers of generalization led him to teach *truths*, ideas and theories far in advance of his day, many of which find their full confirmation in recent discoveries and observations.

Peace to his ashes ! and honor to his memory !

As yet, every interest in our country is youthful and promising. Placed in such a field of activity, with so much to do for city, country, humanity, and God, no one should " sit idly waiting for some noble work to do." Then let us encourage every effort for the education of our people, make useful the arts and sciences, cultivate grains, fruits and flowers, build towns, cities, schools, churches, colleges, and railways. The records of our country—of war, of education, of peace and of life—tell of the noble deeds of those of our own number, who were educated here. The joy of my heart is full on this occasion, in contemplating the changes wrought in this fair land since the year 1836; thankful to see so many of the Alumni present—to have the privilege of meeting in a temple so beautiful. When the science of medicine is taught by learned men—courteous, manly—*their works praise them.* All of these noble men are members with us.

The science of medicine represents all sciences. "The arts are grouped in beauty near with thoughtful science, who their work commands."

Our studies have prepared us for other duties beside those of social life. It is marvelous that

"Immortal genius in one day should cause
Art and science to spring to birth.
Genius pointed her scepter toward the waste
Of hill and dale, and far-extended plain."
The inventor has been busy with his plan
To lighten toil to will of man.
The discoverer has found the pathless way,
As truth divine has led the way.

So that the farmer gathers his golden harvest with machinery; toil is lightened by giant powers.

There is another field of pleasure and usefulness. Nature is grand—comprehensive, a system of many circles, each dependent upon the other—ever so minute and delicate in grade—with harmony in action in crystals, plants, animals, man:

—"The chain is love,
Combining all below and all above.
Nothing stands alone.
The chain holds on—
With thoughts well freighted,
The chain holds strong embrace."

Our studies qualify us for the examination of the elements, the various forms of life, the manipulations of the world without, as well as the world within us.

To the younger members of alumni, we give you a glad welcome to membership. Despise not the day of small things; little by little, step by step, success will attend you in your enterprise. It is early morning with you; hence, arise and shine, and make your own summers.

"Pleasant thoughts in the day dream of life,
When youth and ambition join hands for the strife."

You have drank at the crystal fountain. You are now qualified to join in the march of fame. If you are good anatomists, you will soon become good physiologists, pathologists and surgeons. Never permit yourselves to become "rusty" in your knowledge of anatomy. When you prescribe a dose of medicine, do it in accordance with your understanding of the laws of chemistry and the laws of life. To become proficient in your calling, pay strict attention to your books. You will find great pleasure in your study of physiology. There is no scene that will gratify you more than that when you behold those mysteries of life. Be cautious in your conclusions, and modest in your opinions. Consult your books often; they are your friends. We would have you to be good citizens; to take active duties in civil life; to take a noble part in any good work that will give character to yourself and your profession.

Changes are pluming mighty thoughts—
Thoughts that wing their way far onward,
Onward to scenes more glorious.

If you have an opportunity, teach the young; such opportunities offer in graded schools. Be always ready to assist in regulating civil society; in establishing schools, seminaries, churches; and in caring for the unfortunate. Please to ennoble labor in nature's plan. It should be virtue's brightest diadem. Speak kind words to the new beginner in our profession, and support those who are just entering upon the profession. This is the golden rule:

Walk erect, like men.

So conduct yourselves as not to become involved in political strifes—sectarian animosities. Demean yourselves manly to the young and old—to the rich and poor—to all men. Have mercy, have charity, have liberal views, that you may bless all, and God will bless you.

You are about to take the first steps that will form your character. Every act—the manner you spend your time—the attention you give your profession and your home, will form your character. You will stand as representatives of the medical profession. Then, be careful—let your demeanor be just, liberal, wise, kind and professional.

If you err, perhaps it will be for a want of a proper division of your time. Have a time for every duty—use little moments with strict economy. The little savings will make you rich. Keep notes of your cases. Give yourself a clinic on every case you have. If an epidemic prevails, make notes of all of its peculiarities—report important cases—your inventions and your discoveries to older minds, previous to publication. Such faithful labor will lead you to wealth, to honor, and to a good reputation.

You have been honored with graduation, and received as members of alumni. Your aspirations have begun already to mount up—anxious to take active duties.

With pride, the older members have received you as members of this Association, and with pride we can commend you to your calling.

Before you is your life—the rainbow of hope is spanned. Will your pathway be pleasant and profitable? or will it be beset with trials, dangers and temptations?

Our education, above all others, fits us for social life, for a fine appreciation of love, of virtue, of honor, of integrity—for a large and a comprehensive view of the duties of life that elevates the mind.

Dear friends, there is a lion in the way—*insobriety*. Good and noble men have been wounded by him. He is a monster—a dragon. If thine eye is not dim, behold the moral and physical destruction made on the human race. That happy family, whose hearts were as buoyant as the morning breeze, and as happy as the bird that flits thereon—a change is wrought. Their souls are now chafed and deranged. Around that sublunary haven, where once gently wafted the soft and spicy breezes of true love and friendship, the atmosphere is now glistening with the chilly dews betokening the approach of winter. No sunny face or sparkling eyes greet you. Children are there taught the broad road to sin. If thine ear is not heavy, where once was heard the prayer of praise, is now cursing; where once the notes of joy were sung, and the harp notes stealing, is mourning, sobbing, and crying; and where once the trowel of devotion spread liberally the cement of love and harmony, now angry and discordant passions reign as the tyrant of the realm, scattering confusion, disease, misery and death. Shipwreck is made of virtue, of hope, and of joy. Misery is in the post of peace.

Be assured, a perverted pleasure or principle produces a corrosive trouble.

Gentlemen: We have taken an impressive survey of the achievements of the past, and viewed them in their blended lights and shades, and seen that the small town has become a marvelous city. The vast, uncultivated prairies now yield plentiful harvests, wealth and commerce active, and medical science developing new truths. Medical men have caused the lamp of knowledge to send forth its fair rays of light to shine away the clouds of error and superstition, that ignorance and human woes might give place to health, peace and happiness. Its light beams brighter, revealing the true economy of life.

> Around the man of learning,
> The arts and sciences at command,
> To lay richest treasures at his feet.

Let us see to it that the honor, the reputation and the usefulness of our Alumni are on the side of genius. That the true light that shineth away the darkness, that guides the intellect in the way of invention and of discovery, and enables the mind to bring into use the elements for the support and protection of our race.

You will soon return to your fields of labor.

> Home is the dearest place on earth;

Make yourselves pleasant homes; ornament them with fruits and flowers—

> " With margin green and beautiful,"
> With roses and nodding lilies,
> To gladden the sunlight of smiles.

In your pleasant homes, forget not to cherish in your minds in a becoming manner a true love for " Rush."

> " From the heart's sunshine, a ray often mounts,
> To break on the lips with a smile;
> Many a word from the lips we love much,
> Strikes a chord of love's true emotion."

Ever will it be my prayer that God will bless this temple, this college, its graduates, and all true members of our noble profession everywhere.

When time with us is no more, may one and all be admitted to the banquet and the joys of heaven.

